# Sex-specific effects of LiCl treatment on preservation of renal function and extended life-span in murine models of SLE: perspective on insights into the potential basis for survivorship in NZB/W female mice

**DOI:** 10.1186/s13293-016-0085-7

**Published:** 2016-06-27

**Authors:** David A. Hart

**Affiliations:** Department of Surgery, Wound Healing Initiative, McCaig Institute for Bone and Joint Health, University of Calgary, 3330 Hospital Drive NW, Calgary, Alberta T2N 4N1 Canada; Faculty of Kinesiology, University of Calgary, Calgary, Alberta Canada; Centre for Hip Health and Mobility, University of British Columbia, Vancouver, British Columbia Canada

**Keywords:** Murine SLE, Preventing renal failure, Lithium treatment, Sex differences, Females

## Abstract

Considerable research effort has been invested in attempting to understand immune dysregulation leading to autoimmunity and target organ damage. In systemic lupus erythematosus (SLE), patients can develop a systemic disease with a number of organs involved. One of the major target organs is the kidney, but patients vary in the progression of the end-organ targeting of this organ. Some patients develop glomerulonephritis only, while others develop rapidly progressive end organ failure. In murine models of SLE, renal involvement can also occur. Studies performed over the past several years have indicated that treatment with LiCl of females, but not males of the NZB/W model, at an early age during the onset of disease, can prevent development of end-stage renal disease in a significant percentage of the animals. While on Li treatment, up to 80 % of the females can exhibit long-term survival with evidence of mild glomerulonephritis which does not progress to renal failure in spite of on-going autoimmunity. Stopping the treatment led to a reactivation of the disease and renal failure. Li treatment of other murine models of SLE was less effective and decreased survivorship in male BxSB mice, exhibited little effect on male MRL-lpr mice, and only modestly improved survivorship in female MRL-lpr mice. This perspective piece discusses the findings of several related studies which support the concept that protecting target organs such as the kidney, even in the face of continued immune insults and some inflammation, can lead to prolonged survival with retention of organ function. Some possible mechanisms for the effectiveness of Li treatment in this context are also discussed. However, the detailed mechanistic basis for the sex-specific effects of LiCl treatment particularly in the NZB/W model remains to be elucidated. Elucidating such details may provide important clues for development of effective treatment for patients with SLE, ~90 % of which are females.

## Background

In autoimmune diseases, immune dysfunction can lead to the induction of autoantibodies and/or self-reactive lymphocytes such as T-lymphocytes (reviewed in [1, 2]). In some autoimmune diseases, such as myasthenia gravis, the target of the autoantibodies are very specific for particular tissues (reviewed in [3]), while in others such as rheumatoid arthritis, several or a few joints can be involved, but it is a systemic disease. Similarly, in systemic lupus erythematosus (SLE), a spectrum of autoantibodies can be present and a number of target tissues (e.g., kidney, skin, other organs) may be involved (discussed in [4–6]). As with many autoimmune diseases, the incidence of such diseases is much greater in females than males, with the ratio in SLE being ~9 F/1 M (reviewed in [7]).

With diseases such as SLE, it is clear that there is a genetic component to the disease (reviewed in [8]), as well as some ethnic associations (reviewed in [9–12]), particularly with end-stage renal dysfunction. Thus, many African-Americans have serious progressive renal involvement compared to non-African-Americans.

While patients with SLE can have a number of target tissues involved, the kidney is a major organ that is involved. However, in spite of a somewhat similar pattern of autoantibodies being present, some patients can have very modest glomerulonephritis (class I/II) which does not progress while others can have severe renal involvement which progresses rapidly to tubulosclerosis and end-stage renal failure (class III/IV) (discussed in [13, 14]). Details regarding the basis (e.g., genetics, immunological dysfunction, target organ responsiveness) for such variation in renal involvement remain to be elucidated. What is clear is that in some patients, the target tissue (e.g., kidney) is somewhat protected from end-stage damage, while in others, this is not the case. Some of these differences in human disease appears to have a genetic basis with kidney-associated genes playing a role (reviewed in [15, 16]). While considerable effort has gone into trying to understand the immune dysfunctions leading to renal involvement with many new treatments (discussed in [14]), our understanding of target organ resistance to immune insults is less well defined. However, murine models of SLE can likely provide useful information regarding human disease (reviewed in [17, 18]).

### Murine models of SLE

There are several mouse models of SLE available for study (reviewed in [19–21]). Some of these, such as the MRL-lpr or BxSB mice, have defined mutations that contribute to the autoimmune disease. For instance, in the BxSB model, only males get the disease, and in the MRL-lpr mouse, the parent strain (e.g., MRL-n) has a number of immune dysfunctions which contribute to the development of the autoimmunity and includes excessive lymphoproliferation in the liver and spleen (reviewed in [19, 20]; and discussed in [22]), factors not usually observed in patients with SLE. In contrast, in the NZB/W F1 model both parental strains contribute to the SLE-like disease, and a number of genes are involved. Some of these relate to the immune dysfunctions which appear in the F1 mice, while others appear to be related more to the target organ involvement. In this NZB/W F1 model, females get a more rapid and progressive disease with renal failure by ~10–12 months of age, while the males get a much more mild disease and do not start to experience overt renal failure until ~20+ months of age (reviewed in [19, 20]). Thus, some sex-related factors (e.g., sex hormones; discussed in [23, 24]) also appear to play a role in development and progression of end-stage renal failure, possibly at the level of sex hormones on immune activities. Therefore, whether sex hormone variables may be related to immune dysfunction activities or some other factors related to the kidneys themselves is still not clear, but this point is relevant to the discussion which follows. Interestingly, females of the NZB/W F1 combination gain significant weight as they age, often leading to weights that are double or more those of the parent strains [25, 26]. Much of this weight is due to subcutaneous and intraperitoneal fat deposition. Given parallels between target organ involvement, and the sex differences noted above, the NZB/W F1 model was chosen for detailed investigation regarding treatments that could prevent renal failure and, thus, prolong the life-span of animals in the face of immune dysfunction and autoantibodies.

### Influence of lithium chloride treatment on disease progression in male BxSB, female MRL-lpr, and female NZB/W mice

#### Background

Salts of Li have been used extensively in the treatment of bipolar disease since ~1949 when Cade reported that Li treatment of guinea pigs led to a calming effect [27]. Interestingly, low concentrations of Li salts are known to be a component of a number of springs touted as being beneficial for treating a variety of ailments. However, use is often associated with side effects (reviewed in [28]).

Another limitation of Li salts in the treatment of bipolar disease is that efficacious doses are close to toxic doses, and thus, patients taking Li salts must be carefully monitored and evaluated for side effects. As Li salts are natural compounds, they cannot be patented as such, so there is little incentive for industry to invest in their applications. Li exists as two major isotopes, Li-6 and Li-7, and natural Li is ~95 % Li-7. This is relevant since the ionic radius of Li-6 is similar to potassium, while that for Li-7 is similar to Mg, and thus, the two isotopes could affect different ionic environments.

Li salts are known to influence adenyl cyclase [29], the NaK membrane transporter [30], and the Wnt/beta-catenin system (reviewed in [31]). While it has been very difficult to identify normal functions of Li in animals, Li can impact a number of biological systems and thus can be considered a regulator molecule (discussed in [32]). In a series of reports in the 1970s, 1980s, and early 1990s, it was demonstrated that Li salts can influence lymphocyte and polymorphonuclear leukocyte (PMN) activities both in vitro and in vivo (reviewed in [32–34]). Therefore, it was possible that Li treatment could have immunomodulatory influences on immune processes.

While not used in SLE per se, it has been noted that patients on Li salts who also had SLE sometimes experienced an exacerbation of their SLE, but this was not a consistent finding (discussed in [32]).

#### Studies of Li effects on survival in murine models of SLE

Initial studies investigated the effect of 4 mg of LiCl/day via the intraperitoneal (IP) route on survival of young male BxSB mice, female MRL-lpr mice, and female NZB/W mice. Li treatment led to decreased survival in the BxSB model [35], some modest enhancement of survival in MRL-lpr female mice, depending on how early the Li treatment was initiated [22], and significant survival of female NZB/W mice [25, 26].

In the NZB/W model, survival was significantly prolonged by >50 % when the female animals were started on Li treatment at 8–10 weeks of age, an age when the SLE was detectable (e.g., autoantibodies in serum, early glomerulonephritis) [25, 26]. This enhanced survival in a subset of female NZB/W mice was a “cure” but was not a definitive cure as stopping Li treatment led to a reactivation of renal disease and subsequent accelerated death. Therefore, treatment with Li led to the transient resistance to terminal end-stage renal failure in a subset of female NZB/W mice with early disease. Why this particular subset of female animals survived while on treatment, and others remained susceptible to the autoimmune insults, was unclear from these initial studies. However, histologic analysis of kidneys from some survivors indicated that they expressed signs of continued glomerulonephritis but apparently failed to convert to tubule sclerosis which is critical in the development of renal failure. In this regard, these survivors resembled some human SLE patients who have evidence for glomerulonephritis class I/II which does not progress to tubule involvement and sclerosis. Relevant to the current discussion, female NZB/W mice on Li did develop polydipsia while being treated, so renal function was impacted in these mice. Interestingly, polydipsia developed in all mice treated with Li salts, so this was not unique to survivors.

### Additional studies to further characterize the effectiveness of LiCl treatment in NZB/W mice

#### Timing of initiation of treatment

Subsequent studies indicated that there was an age dependence for the effectiveness of LiCl treatment in female NZB/W mice. The optimal time to initiate treatment was found to be 6–8 weeks of age, when features of SLE were just beginning. Delaying initiation of treatment to 16 or 28 weeks of age led to a diminished number (28–35 %) of long-term survivors [36]. It was also found that initiating treatment at a very early age of ~4 weeks of age did not further enhance survival over that observed at 6–8 weeks of age (~40 % at 4 weeks of age vs 50–60 % at 6–7 weeks of age) [36]. In fact, starting Li treatment at 4 weeks of age actually led to a somewhat lower long-term survival rate. Thus, optimization of effectiveness of LiCl treatment was potentially associated with events occurring between 4 and 6–7 weeks of age, with a ~60 % long-term survival rate. The most prominent event, with associated secondary changes occurring in mice during this time frame, is likely the onset of puberty. Puberty in mice occurs at ~6 weeks of age (discussed in [37]). As the cells in the kidneys do express estrogen receptors (reviewed in [38]), they should be responsive to sex hormones. If this association between the onset of puberty and optimal LiCl responsiveness is correct, the timing results likely indicate that Li modulates puberty-associated changes in gene/protein expression/location in some manner, rather than prevents the expression of some tubule-associated gene/protein expression.

#### Effect of isotope, dosage, timing, and route of exposure on Li effectiveness

Natural Li exists as predominantly two isotopes, Li-7 and Li-6 (discussed in [21, 32–34]). Natural Li is comprised of ~95 % Li-7 and ~5 % Li-6. This point is potentially relevant to understanding the mechanism(s) of Li action as Li-6 has an ionic radius similar to Na, while Li-7 has an ionic radius more similar to Mg (discussed in [21, 32–34]). Therefore, Li-6 may affect NaK ATPases more readily than Li-7. In contrast, Li-7 may affect Mg-dependent systems more effectively (e.g., adenyl cyclase). Daily treatment of female NZB/W mice starting at ~8 weeks of age with 4 mg Li-6 (95 %) or Li-7 (99 %) led to similar survival rates over the long term [25]. Thus, these results did not provide additional insights into potential mechanistic effects of these Li isotopes. However, it was noted that Li-6 did induce more polydipsia that did Li-7 (Hart, unpublished observations).

Administration of the LiCl via the drinking water was ineffective in promoting long-term survival of female NZB/W mice at doses that were not toxic [39]. Interestingly, Li-6 in the drinking water was much more toxic than Li-7 at the higher doses investigated [39]. Administration of the Li via the IP route was only slightly effective when 2 mg/day was used instead of 4 mg/day, as assessed by long-term survival [40]. Interestingly, administering 4 mg LiCl/day via the IP route was partially dependent on the time of day the Li was administered, with administration during the morning more effective than the evening [40]. As mice are more active at night, and their adrenal steroid pulses are the reverse of humans (e.g., higher in the morning and lower in the afternoon), higher levels of glucocorticoids occur in the evening. Interestingly, administration of melatonin did not mimic the effectiveness of Li [40].

Given that administration of 4 mg LiCl in the morning or evening was effective in protecting a subset of female NZB/W mice from end-stage renal failure, studies were undertaken to treat a panel of 6–8-week-old females with 4 mg LiCl in the morning and evening (a total of 8 mg of Li/day). Animals were treated 7 days/week for 18 months [41]. This treatment regimen led to the long-term survival of ~80 % of the mice to a time when 100 % of the control mice had been dead for >7 months [41]. Again, these survivors exhibited some evidence of glomerulonephritis, but no tubule sclerosis ([41], and unpublished observations). Furthermore, cessation of Li treatment at 18 months of age again led to a reactivation of the disease and subsequent death. Thus, even with this more intense treatment regimen, one of the major target organs was only protected during treatment, and following stoppage of treatment, the target organ was again apparently susceptible to immune insults. The impact of Li is a reversible phenomenon even after a protracted treatment protocol. Thus, while on the LiCl treatment, the vast majority of the NZB/NZW females were “cured” of the development of end-stage renal failure, but this cure was conditional and not definitive.

#### LiCl treatment does not enhance survival of male NZB/W mice

As discussed above, male NZB/W mice exhibit a more mild disease course than females (reviewed in [19, 20]). The onset of the SLE is slower in males, and they live a much longer life-span. Treatment of male NZB/W mice with 4 mg LiCl/day IP, 7 days per week starting at 8–10 weeks of age, did not influence survival out to nearly 81 weeks of age, a time point when ~90 % of the untreated controls had died [36]. Therefore, the disease course must be somewhat different in the females where the SLE is more aggressive.

The above findings with male NZB/NZW mice, and the earlier discussion of the effectiveness of LiCl treatment increasing following onset of puberty, lead to the possibility that androgens are protective against development of renal dysfunction and estrogens contribute to the susceptibility of female NZB/NZW mice to renal dysfunction. Such conclusions are supported by the literature, where it has been noted that castration of male NZB/NZW mice increases their susceptibility to such renal disease and ovariectomy of female mice leads to a diminished susceptibility (reviewed in [19, 20]). Thus, future studies assessing the LiCl responsiveness of castrated males and ovariectomized females could provide further clues to the renal mechanisms responsible for responsiveness. Of particular interest would be comparison studies with NZB/NZW mice castrated or ovariectomized prior to onset of puberty or after puberty onset.

#### LiCl treatment does not overtly alter autoantibody profiles in female NZB/W mice

As discussed above, Li ions have been reported to exert some immunomodulatory effects (discussed in [21, 32–34]). To investigate whether Li treatment of female NZB/W mice led to overt immunosuppression, serum from treated and untreated mice were analyzed for autoantibodies, antibodies to porcine renal tubule cell lysates [42], and RNA from treated and untreated mouse kidneys were assessed for TNF transcripts [43].

To assess the effect of Li treatment on autoantibodies, female NZB/W mice were started on 4 mg Li-7 at 8 weeks of age. Serum levels for anti-single stranded DNA and anti-gp70 were not significantly different between treated and untreated mice at 16, 22, and 28 weeks of age (prior to renal failure). Western blotting analysis of serum reactivity to porcine renal tubule lysates from treated and untreated mice also did not reveal any group-specific differences, although some individual differences were noted within both groups [42]. Similarly, messenger RNA (mRNA) levels for the proinflammatory cytokine, TNF-alpha, were similar in extracts of total kidneys of treated and untreated mice at 28 weeks of age [43]. While the analysis was not extensive, and did not evaluate a large number of autoantibodies or cytokines, no evidence for an overt immune effect of Li treatment was detected. However, it is clear that technologies have changed since these studies were performed and the approach should be revisited to gain a more complete perspective regarding Li effects on immune parameters.

#### Influence of Li treatment on gene expression in the kidney

As mentioned above, mRNA levels for TNF-alpha were not inhibited by Li treatment in kidneys of Li-treated female mice. Additional analysis also revealed that expression of gp70, a reported nephritogenic stimulus [44] in female NZB/W mice, was not inhibited by Li-treatment at 16, 22, and 28 weeks of age [43]. In fact, at 22 weeks of age, mRNA levels for gp70 were significantly elevated in Li-treated mice.

Interestingly, mRNA levels for the plasminogen activator, urokinase, were significantly elevated at 16 (*p* < 0.01), 22 (*p* < 0.00001), and 28 (*p* < 0.01) weeks of age. As urokinase is produced by tubule epithelial cells (reviewed in [45]), and expression could interfere with fibrin deposition and development of tubule sclerosis (discussed in [43]), this finding may be relevant to the mechanism of Li action. However, in vivo, it may be more complicated than that interpretation since elevations in urokinase mRNA levels were somewhat uniformly elevated in mice at those ages, but earlier studies had indicated only about 50 % of the mice would be long-term survivors [25]. Therefore, elevated urokinase mRNA levels were associated with Li treatment, but that association was likely not a complete explanation for long-term survival in this model.

While not a complete explanation, this finding regarding urokinase is interesting and it may be a contributing factor in survivorship in these Li-treated mice and assist in protection against loss of tubule integrity. Urokinase is produced by convoluted proximal tubule cells and the thick ascending limb of Henle’s loop [46]. Tubule epithelial cells also express receptors for urokinase, a finding that may also be relevant for the localization of the urokinase (discussed in [45]). Therefore, there is associated evidence that urokinase could be involved in long-term renal protection, but this proteinase cannot explain the fact that only a subset of Li-treated mice are long-term survivors as all mice on Li treatment exhibited elevated urokinase mRNA levels.

As mentioned above, all Li-treated female NZB/W mice developed polydipsia, and so these associated changes also do not explain the ~50 % long-term survivors when treated with 4 mg LiCl per day [25]. Interestingly, Rojek et al. [47] have shown in rat models that a number of renal genes are expressed differently following lithium treatment and induction of polydipsia. Therefore, this approach should likely be repeated with NZB/W female mice +/− Li treatment to gain further insights in this model. Other investigators have also advanced the concept that Li is an excellent tool to study renal physiology and biochemistry (reviewed in [48]).

Interestingly, studies using isolated porcine proximal tubule cells in vitro have indicated they are very resistant to damage by Li [49]. In contrast, Li treatment of porcine distal tubule cells in vitro lead to apoptosis of this porcine cell line (PK (15)) [50]. Thus, different cell populations in the kidney may be responding to Li exposure in a heterogeneous manner. Whether this resistance of porcine proximal tubule cells can be extended to the analogous murine cells is not known. Unfortunately, in the autoantibody studies described above (42), we used porcine cell line (PK (15) and LLC-Pk1) cell lysates but did not use Li-treated porcine tubule cells for comparison to assess whether Li treatment led to alterations in detection of specific protein targets. Such further studies in pig models may lead to valuable information in this regard.

However, these findings in Li-treated NZB/W mice could be revisited using improved molecular technologies to better quantify the molecular and cellular changes occurring and to investigate the potential underlying mechanisms leading to a long-term survivorship while on Li in more detail. Perhaps, focusing on lithium-mediated effects on G-protein signaling [51], or molecules such as e-selectin [52] which contribute to macrophage adhesion and subsequently, participates in lupus nephritis [53]. Relevant to this discussion is the fact that regulation of e-selectin expression involves the GSK-3 pathway, a pathway that can be regulated by LiCl [54, 55].

### Possible molecular mechanisms underpinning Li action in NZB/W female mice

#### Potential direct effects of Li on the kidney

As mentioned above, Li treatment led to induction of polydipsia in all mice, and as this is likely due to an effect on collecting tubule ducts, this is evidence of a direct impact of Li on these cells. Other in vitro studies examining the effect of Li salts on porcine renal tubule cells revealed that Li induced apoptosis in the PK(15) tubular cell line [50], but the porcine proximal tubule cell line LLC-PK-2 was very tolerant to similar concentrations of Li [49]. While these two cell lines have been adapted to growth under in vitro conditions, the findings do potentially indicate that tubule cells derived from different parts of the kidney may respond to Li differently.

At the molecular level, as discussed above, Li can influence a number of intracellular pathways, and presently, it is not clear which one(s) are affected by in vivo treatment of the NZB/W mice. Of particular interest is the glycogen synthase kinase-3 (GSK-3) pathway which has become important in a number of systems (reviewed in [54, 55]) and the Wnt and beta-catenin pathways discussed earlier. LiCl is known to inhibit renal GSK-3 activity in C57Bl/6 mice [55]. Perhaps, with the availability of a large number of inhibitors for several potential Li targets becoming commercially available, this could be revisited to gain more detailed understanding of potential direct influences of Li on renal cells.

#### Potential indirect effects of Li

Interestingly, as a consequence of the LiCl treatment of female NZB/W mice, they failed to gain weight and, specifically, failed to deposit the usual subcutaneous and intra-abdominal fat [25, 26]. The lack of fat deposition was complete and the mice closely maintained their starting weights. Once treatment was stopped, the surviving mice again started to deposit fat. The basis for this fat deposition is unknown and its relationship to the SLE symptoms and disease progression also unknown. At the time the studies were performed, the observation was interesting, but how it could be associated with the renal function and immune parameters was not overtly evident. However, it is reported that obesity is associated with the development of a number of autoimmune diseases [56], so there may also be linkages between progression of disease via secondary sequelae (e.g., renal involvement) and obesity.

However, it has been reported that caloric restriction of female NZB/W mice leads to a decline in cytokine expression and disease progression irrespective of dietary fat [57]. More recently, a focus on obesity has revealed that obesity is associated with development of a metabolic disease, accompanied by a systemic inflammation (discussed in [58, 59]), as well as the other impacts of obesity on the kidney [60]. Furthermore, such metabolic disease may be a risk factor for renal disease (discussed in [58, 59]). Therefore, it may be necessary to return to the mechanisms of fat deposition in this model and the consequences of it as the SLE-like disease progresses, as it may also be a contributing factor in the disease process and the renal response to Li. However, Li treatment affected fat deposition in 100 % of the NZB/W when treatment was started at 8–10 weeks of age, a time when only ~50 % of the mice were destined to be long-term survivors. Thus, the fat deposition aspect of the Li treatment may be only a contributing factor, and secondary to some as yet, undefined specific target response. However, while an impact on diet-related factors may not account for all of the Li effects on NZB/NZW female mice, it could be a regulatory component of the impact which allows or permits some other Li-specific target(s) to exert a strong impact on survival.

Thus, Li effects on disease progression in NZB/NZW female mice may relate, in part, to what the animals are fed or their nutritional status. Mice housed in a university vivarium are usually fed a standard chow diet that is commercially available (most are casein- or corn-based diets), a diet likely not very similar to what a mouse would subsist on in the wild. This scenario sets the stage to ask whether Li treatment was affecting, in as yet unidentified manners, the impact of this artificial diet on the immune system, the kidney, an obesity-induced altered inflammatory state, and/or even the mouse microbiome [61] of these susceptible NZB/NZW mice.

Relevant to this discussion is a report by Chandrasekar and Fernandes [62] which indicated that NZB/NZW female mice fed a diet rich in omega-3 lipids exhibited decreased pro-inflammatory cytokine expression, as well as increased antioxidant gene expression. The treated mice had an extended life-span, similar to the mice treated with a single dose of Li/day starting at the age of 8 weeks. However, in the Chandrasekar and Fernandes studies, the mice on the omega-3 rich diet weighed the same as the untreated mice [62]. Therefore, Li treatment could be impacting the negative influence of the chow diet on disease development and progression, in part like the omega-3 rich diet, but with some additional metabolic targets, possibly mediated by glycogen synthase kinase-3 beta (GSK-3beta; [54, 55]). Interestingly, a number of reports in a variety of murine lupus models [63–67], including the MRL-lpr model and the NZB/NZW model with females, have supported the concept of dietary influences on disease development and progression, including nephritis. Therefore, Li effects on long-term survival may involve multiple targets due to its range of metabolic influences.

Of note, it has been reported that obesity is common in patients with SLE (discussed in [56]). If this possibility proves to have validity from future investigations, then it may require stepping back and re-evaluating potential inductive and exacerbating signals for SLE development and progression.

### Could genetics play a role in the observed protection of female NZB/W mice with lithium?

In humans, it is clear that the genetics are complex and likely include contributions from kidney-related genes [15]. It is also clear that NZB/W mice are an F1 between the two parental strains (NZB and NZW) and that both parental strains contribute genes to the F1 SLE phenotype (reviewed in [19, 20] and others). Thus, the finding that ~50 % of the female NZB/W mice were long-term survivors after initiation of treatment with 4 mg LiCl per day at 8–10 weeks of age raised the potential interpretation that one half of the mice expressed some genetic susceptibility from one parent vs the other. However, increasing the dose of LiCl to 2 × 4 mg/day leads to long-term survival of ~80 % of the mice [41], and therefore, the higher dose appeared to overcome some limitations of the 4 mg dose, findings which would tend to temper the interpretation that long-term survival was associated with the genetics of one parent vs the other.

To begin to better understand some of the potential strain-specific effects of LiCl treatment, the response of a number of commercially available mouse strains (eight strains from Jackson Laboratories), including the parental strains (NZB and NZW), to LiCl treatment was undertaken. The induction of polydipsia in some strains was induced by lower LiCl concentrations than others [68, 69], and in some strains, polydipsia was not overtly induced even by 4 mg LiCl/day. Of particular interest was the finding that induction of polydipsia in NZB and C57BL/6 mice occurred at low doses, while higher doses were required for NZW and BALB/c, and the modest doses were apparently toxic for A/J mice. The response of DBA and C3H/HeJ mice was intermediate to that of the NZB and C57BL/6 mice. What is very interesting from these data is the fact that the NZB and C57BL/6 mice are black and all of the low/negative responders had a white coat color. Furthermore, albino C57BL/6 mice were still responsive to Li induction of polydipsia, so that the coat color contributions were likely not directly involved in polydipsia induction (Hart, unpublished observations). Interestingly, tyrosine hydroxylase has also been reported to not be associated with lithium responsiveness in patients with affective disorders [70]. Furthermore, African-Americans and Hispanics more responsive to low-dose lithium than are Whites [71]. Therefore, the response patterns to LiCl in mouse strains may provide some insights to effectiveness in humans, but it is not yet clear whether there will also be sex differences in this regard.

Other investigations using very different readouts (e.g., brain functioning) have also found somewhat similar results regarding coat color [72]. In those studies, C57BL/6 and Black Swiss mice were Li-responders, but CD-1 and NIH Swiss mice (e.g., white coats) were non-responders to Li treatment. Again, C3H/HeJ mice were modestly influenced by the Li treatment. Therefore, the relationship between coat color and lithium effectiveness is likely a more general phenomenon but could have impacted the responsiveness of the BXSB and MRL-lpr mice in addition to sex!

The common finding of black coat color being relevant to Li responsiveness is very interesting and may indicate that in the studies detailed with NZB/W F1 mice, and in spite of the above discussion regarding long-term survivorship, Li may be primarily influencing genes provided by the NZB parent, but this speculation remains to be confirmed.

Interestingly, treatment of female NZB/W F1 mice with alpha-melanocyte stimulating hormone did not provide any significant renal protection, so that hormone is likely not involved in long-term survivorship while on Li [73]. In contrast, alpha-MSH is reported to ameliorate SLE-like activity in the pristine-induced mouse model [74]. Furthermore, alpha-MSH has been reported to have potent anti-inflammatory activities and protect against acute renal failure in rodent models, possibly by direct effects on renal tubules via melanocortin receptors [75]. Therefore, the inability of alpha-MSH to protect against renal failure in the female NZB/NZW model is at odds with some of the literature regarding alpha-MSH and acute renal insults and may be due to the chronic nature of the SLE-mediated effects or perhaps due to other non-melanocortin receptor associated events.

As the genetic basis for mouse coat color and human pigmentation is very complex [76–81], the relationships to renal functioning after Li treatment may be very complicated as well, particularly given the sexual dimorphism observed for Li treatment in the NZB/W mice. However, there may be some “common ground” for some of the observations related to Li affects on the females. That is, the melanocortin system may be involved in coat color, as well as obesity (reviewed in [77, 81]) and the renal system (discussed in [80]). Interestingly, mutations in the melanocortin-4 receptor are associated with early onset obesity in humans and in mice [81]. Therefore, it is possible that Li may be affecting elements of a single, as yet undefined, system to modify the kidneys of these mice, as well as the loss of fat that was observed.

### Additional considerations regarding why LiCl treatment is effective in female NZB/W mice

In the NZB/W model, as discussed above, it is known that subjecting females to ovariectomy leads to an inhibition of disease development and progression (reviewed in [19, 20]), while castration of males leads to an enhancement of the disease progression (reviewed in [19, 20]). However, whether this is due to immune variables or renal variables cannot be ascertained in detail. Interestingly, the association of Li effectiveness with the onset of puberty, and the lack of effect in males, may indicate that the targets of Li treatment are related to either sex hormone modifications to the kidney or as yet undetected immune dysfunction, and the current findings are consistent with the above information. Interestingly, the presence of two X chromosomes vs a XY complement has been reported to impact the onset and progression of disease on a pristine-induced lupus model [82]. Furthermore, whether the cells in target tissues express estrogen receptor alpha or beta (ER-alpha or ER-beta) may also play a role in gene expression (discussed in [83–85]) via hormone response elements (complexes of ER + hormone) or via formation of complexes with other transcription factors (discussed in [83–85]). Thus, ER can regulate gene expression alone, as well as in complexes with hormone. Interestingly, estrogen receptors in NZB/W mice are reported to be variants compared to those in non-SLE mouse strains (reviewed in [86]). Thus, regulation of gene expression by such variants may predispose for SLE development and progression. Therefore, the “how” of Li effects is still not clear assuming there is a direct link between the kidney and Li, in addition to other potential targets.

Much of the above discussion has focused on the “how” LiCl treatment could be influencing female NZB/W mice, but the real question relates to the “why” behind the observations! The why may also relate to preparing the kidney for pregnancy-related events (e.g., mineral retention for a successful pregnancy, specific cell-type alterations to retain nutrients during pregnancy to transfer to the offspring, or an increased demand on general renal function due to carrying large litters (e.g., control of hypertension), or other female-specific activities related to successful pup survival (e.g., lactation)). Such speculations must await further investigation, but such possibilities can be addressed using modern molecular, biochemical, and genetic tools.

### Are the Li treatment of NZB/W female mice findings relevant to human SLE?

The other important questions of the “why and how” issues relate to extrapolation of the mouse findings to humans. These are critical questions as considerable grant funding has gone into understanding these mouse models, with the hope that the findings are relevant to the human condition. In humans with SLE, ~90 % of patients are female, but the incidence of nephritis is higher in the population of males than in the females [87–90], but ethnic variables (black, white, Asian) appear to also play relevant roles in the sexual dimorphism [91–93]. Interestingly, males are often older when the disease is diagnosed, and some of these men have low sex hormone levels.

Thus, by numbers alone, there are more females with lupus nephritis than males, but even in the small percentage of males, it is common and can be more severe [9, 86–93]. Therefore, development and progression of lupus nephritis is either mechanistically different in males and females, or the findings in NZB/W mice cannot be readily extrapolated to humans and thus are restricted to mice with a specific background/genetic makeup (e.g., they are mice after all with millions of years of optimizing their regulatory systems for survival of the species). There are however some areas of concordance between murine coat pigmentation with human skin color, as in human patients with SLE, there appears to be more renal involvement in non-Caucasians than Caucasians (discussed in [9, 88, 93]). Thus, in Caucasians, there may have been some loss of function associated with skin color during evolution that did not compromise survival of that subset of humans. Therefore, whether the “why” of the findings with LiCl and NZB/W mice can potentially be translated to humans remains to be determined, but using comparative systems biology approaches, additional commonalities may be elucidated.

#### Summary

Li treatment has been shown to influence disease course in murine models of SLE for females, but not males. In particular, the response of female NZB/W mice to Li is dramatic from the perspective of survivorship and maintenance of renal function. Li treatment has been shown to alter a number of parameters at the level of the kidney (e.g., prevention of renal sclerosis, induction of urokinase, and induction of polydipsia), but these have not been directly linked to long-term survivorship while on Li. Indirect effects of Li (e.g., inhibition of fat deposition) were also shown but again were not directly linked to long-term survivorship. While no overt influences of Li treatment on autoimmunity were detected, the spectrum assessed in this regard was limited. Therefore, a number of direct and indirect influences of Li treatment on female NZB/W mice were detected, but it is not yet known if and how such changes are related to long-term survivorship. Long-term survivorship could be dependent on the constellation of changes observed rather than a single alteration, or the constellation of changes could facilitate the effectiveness of as yet known effects of the Li to account for the percentage of female NZB/W mice exhibiting long-term survivorship while on Li.

## Conclusions

The above discussion has provided evidence that Li treatment of murine models of SLE yields varied outcomes. However, the impact on females of the NZB/W F1 strain is profound, and treatment protects a large percentage of the treated animals from end-stage renal failure without detectable impact on immune aberrations. While it is possible that Li treatment did exert some influences on autoimmunity in the female NZB/W mice, it is clear that Li treatment modified the impact of autoimmune dysfunction on the kidney, leading to a low-grade glomerulonephritis without conversion to tubulosclerosis and renal failure. This in itself is remarkable and offers researchers opportunity to pursue the investigations to elucidate the mechanisms underlying such target organ resistance. The underlying basis occurring in females and not males is also somewhat remarkable given the fact that in patients, 90 % are female. At the genetic level, it is also clear that one likely has to separate immune dysfunction (e.g., autoimmunity) from end-organ susceptibility, and the studies presented here offer a route to explore such differences in detail.

While there are some parallels between the murine models and patients with SLE (e.g., influence of sex, ethnicity, renal involvement, etc.), it is unlikely that Li treatment of patients with SLE will or should occur, and other aspects of human SLE are different from the murine models. Firstly, humans likely will not tolerate the comparable levels of Li required to elicit a response in the NZB/W mice. The use of Li salts to treat affective disorders in patients, such as bipolar disease, uses concentrations of Li that are close to toxic, and side effects are common. Secondly, patients on Li usually gain weight rather than lose it (reviewed in [94]), possibly indicating that the molecular targets in humans may be somewhat different. Therefore, more detailed study of the effect of Li on protecting end-organ integrity in the NZB/W model may provide important clues as to the molecular basis for the protection, but the tool to achieve the goal (e.g., Li) may not be directly translated to patient populations. However, elaboration of the mechanisms involved in the NZB/W model may provide useful information regarding the complexity of the problem in human populations.
